# Curative pelvic exenteration: initial experience and clinical outcome

**DOI:** 10.11604/pamj.2023.44.170.37182

**Published:** 2023-04-13

**Authors:** Ahmad Ali Uraiqat, Dania Al Nsour, Khaled Mohammad Mestareehy, Mohammad Saleh Allababdeh, Mo´taz Fawzat Naffa´, Muhanad Mohammad Alrabee, Firas Al-Hammouri

**Affiliations:** 1 Colorectal Unit, King Hussein Medical Center, Royal Medical Services, Amman, Jordan,; 2 Prince Hussein Center for Kidney Diseases, Royal Medical Services, Amman, Jordan

**Keywords:** Pelvic exenteration, colorectal, outcomes, complications, survival

## Abstract

**Introduction:**

in patients with advanced primary or recurrent gynecologic, urologic, or rectal cancers without metastatic disease, extensive aggressive surgery such as pelvic exenteration may be necessary for curative intent treatment. This study aims to present the initial experience and clinical outcome of curative pelvic exenteration procedures for advanced or recurrent pelvic cancer in our center.

**Methods:**

a retrospective cross-sectional study was conducted at the colorectal unit at King Hussein Medical Center in Amman, Jordan, between March 2014 and December 2021. All patients who underwent pelvic exenteration procedures were included in this study. Demographic characteristics, type of procedure, completeness of excision, postoperative complications, morbidity, and mortality were analyzed.

**Results:**

a total of 30 patients underwent thirty-one operations. There were 22 females and eight males with a median age of 55 (range 25-86) years. Twenty-six surgeries were for advanced primary and 5 for recurrent malignancies. Twenty-nine operations were performed for colorectal and 2 for gynecological tumors. There were 19 posterior pelvic exenterations, 2 posterior pelvic exenterations with sacrectomy, and ten total pelvic exenterations. Completeness of tumor excision R0 was observed in 21 specimens, incomplete (R1/R2) in 6 specimens. The overall complication rate was 67.7% and 30-day mortality was 16.7%. Ten (33.3%) patients are disease free at a median follow-up of 22 months.

**Conclusion:**

in our study, pelvic exenteration provides above 40% overall survival at a median follow-up of two years. Gaining experience in this type of procedure, a multidisciplinary approach, careful patients' selection, and preoperative counseling will reduce postoperative morbidity and mortality.

## Introduction

Colorectal cancer is the third commonest malignancy and second in terms of mortality worldwide [1]. In Jordan, it is the second most common cancer among women and the third among men according to the latest Global Cancer Observatory (GLOBOCAN) estimation in 2018 [2]. In the United Kingdom, 50% to 64% of the 14,000 newly diagnosed rectal cancers every year will be locally advanced on presentation, and in approximately 10% of cases, primary rectal carcinomas present with tumor invasion into surrounding organs without distant metastases [3]. In the absence of surgical intervention, the prognosis is poor with a 5-year survival of less than 5%, and a median survival of less than 1 year [4]. After curative resection, up to 32% of patients will develop local recurrence of rectal cancer, which if left untreated; means survival is 7 months [5,6]. The introduction of total mesorectal excision (TME) by Heald *et al*. [7], and advances in perioperative treatment regimens such as neoadjuvant chemoradiotherapy and adjuvant chemotherapy have reduced the rate of local recurrence to <8% [8]. This multimodal treatment reduces loco-regional recurrences but the overall survival rate has not been significantly improved. A slightly better 5-year survival rate has been reported in a systematic review by Li Y *et al*. by recent improvements in cancer staging, developments in surgery techniques, and integration of radiotherapy [9].

Patients with primary advanced (PA) or locally recurrent (LR) colorectal cancer can have disabling symptoms, including persistent bleeding, urinary and fecal obstruction, incontinence, and severe pain caused by bony or nervous tissue involvement. Tumors extending outside the TME plane may be suitable for en block resection if a complete (R0) resection is achievable [10]. A clear margins (R0) resection is the greatest predictor of surgical outcome for rectal cancer, and any surgery must be commenced to achieve histologically clear resection margins [11]. Therefore, en bloc excision of the tumor and/or adjacent organs, via pelvic exenteration (PE), is often necessary to obtain a negative surgical margin [12].

Initially, the rates of postoperative morbidity and mortality were high. However, the progress in surgical technique and patient selection, in addition to advances in imaging and radiation technology resulted that PE is now performed routinely at specialized centers, offering patients a chance of long-term survival with acceptable morbidity and quality of life [13,14].

This study aimed to present our experience and assess the surgical results, oncological outcome, and complications of pelvic exenteration for locally advanced or locally recurrent colorectal and non-colorectal malignancies in our center.

## Methods

**Study design:** this is a retrospective cross-sectional study. A medical record of patients who underwent PE for PA or LR colorectal or gynecological cancer at the colorectal unit, King Hussein Medical Center, Amman, Jordan between March 2014 and December 2021 was assessed and reviewed. All patients were followed-up from the day of surgery until July 2022 or until the last visit or death.

**Inclusion criteria:** all patients with PA and LR colorectal cancers or female reproductive organs (non-colorectal) tumors invading the rectum or sigmoid who had PE were included in our study.

**Exclusion criteria:** patients who refused participation in the research, patients with tumors invading S2 and above and/or bony pelvis operated in other centers, or incomplete data were excluded.

**Preoperative and operative assessment:** all patients had a physical examination, colonoscopy, and pelvic magnetic resonance imaging (MRI) to assess resectability, measurement of the serum carcinoembryonic antigen (CEA) level, and contrast-enhanced computed tomography (CT) scan of the chest, abdomen, and pelvis to evaluate distant disease. Biopsy confirmation of the disease was obtained for all patients. All cases were then discussed in a multidisciplinary team meeting to determine optimal treatment. Pelvic exenteration was performed in all surgical fit patients with locally advanced or recurrent colorectal carcinoma with direct invasion or adherence into other organs or tissue structures in the pelvis, or gynecological tumors invading the colon or rectum to achieve R0 resection. However, when surgical margins were threatened, neoadjuvant chemoradiotherapy was considered according to tumor location and type, and previous history of radiotherapy. Pelvic exenteration was divided into total pelvic exenteration (TPE) with/without sacrectomy and posterior pelvic exenteration (PPE) with/without sacrectomy.

Total pelvic exenteration was defined as en bloc removal of the rectum, ±anus, urinary bladder, lower ureters, and internal reproductive organs in females. In cases of sacral involvement at or below S3, a sacrectomy was also performed. Posterior pelvic exenteration was defined as the removal of the female reproductive organs and rectum or colon, sparing the bladder, when a sacrectomy was performed during PPE; this was defined as PPE with sacrectomy. In cases of anal sparing, intestinal continuity was re-established. A diverting ileostomy was performed in all cases after low colorectal anastomosis, and an end colostomy was constructed in cases of abdominoperineal resection. The choice of urinary reconstruction was made after a discussion between the patient and the colorectal surgeon and the urologist and according to preoperative MRI and intra-operative situation. All operations were performed by the colorectal surgeon and a urologist when needed.

**Variables and data measurement:** preoperative and demographics data including age and sex, body mass index (BMI) and American Society of Anesthesiologists (ASA) score, cancer diagnosis, type and staging, co-morbidities (smoking, hypertension, diabetes, pulmonary and cardiac disease), and preoperative laboratory data (complete blood count and renal function tests, and albumin) were collected for analysis. Any neoadjuvant or adjuvant treatments were recorded. Surgical data such as type of surgery and approach, the radicality of excision (R0, R1, and R2), and the use of stoma were also reviewed. Pathology reports including histology, lymph node status, and margins status were also recorded. Complications were reviewed, and graded according to the Clavien-Dindo classification [15]. Readmission rate and causes and mortality within the first 30 postoperative days were recorded as well as reoperation rates and causes. An anastomotic leak was diagnosed clinically and radiologically by extravasation of contrast at the anastomotic site in a CT scan.

**Follow-up:** all patients attended our clinic for standardized follow-ups every 3 months for the first 2 years and every 6 months thereafter. At each visit, we conducted a physical examination, and digital rectal examination, and measured the serum CEA level. Pelvic MRI and CT scans of the chest, abdomen, and pelvis, were performed every 12 months. Colonoscopy at 1 year after surgery, then as indicated by findings. After recurrence was suspected by CT or MRI imaging, it was confirmed by increased fluorodeoxyglucose (FDG) uptake of the lesion on positron emission tomography (PET)/CT and pathologic confirmation by direct biopsy. Loco-regional recurrence was defined as disease recurrence within the pelvic cavity.

**Statistical analysis:** for reporting frequencies, descriptive analysis was used. Categorical variables were described using the mean and median. Kaplan-Meier methods were used to calculate survival rates, and significance was calculated by a log-rank test. Survival rates were calculated from the day of surgery until death or the last follow-up. Statistical analysis was performed using the SPSS program (IBM SPSS, version 20, Armonk, IBM Corp, Armonk, NY).

**Ethical considerations:** written consent was obtained from all patients before the surgery. All patients have been informed about the risks of surgery and the possible complications and mortality. The study was approved by the local ethics committee (local code 39/6/2022) of the RMS, Amman, Jordan.

## Results

**Participants:** thirty patients that underwent 31 surgeries were included in our review during the study period. Their characteristics are summarized in Table 1. There were 22 (73.3%) women and 8 (26.7%) men with a median age of 55 years (range, 25-86 years); 93.3% were non-smokers. The median follow-up time was 15 months (range, 6-65 months). Nineteen patients (63.3%) received neoadjuvant chemoradiotherapy and 22 (73.3%) patients received adjuvant chemotherapy (excluding 8 patients: 5 in-hospital deaths and 3 refused the treatment).

**Table 1:** patients’ demographics, preoperative and operative data analysis

Characteristics	Value (%)
Gender	
Male	8 (26.7)
Female	22 (73.3)
Age (year), median (range)	55 (25-86)
**Smoking**	
Yes	2 (6.7)
No	28 (93.3)
**ASA**	
I	13 (43.3)
II	15 (50)
III	2 (6.7)
BMI (kg/m^2^), median (range)	24.3 (19-35)
**Primary site**	
Rectal	24 (77.4)
Sigmoid	5 (16.2)
Uterine	1 (3.2)
Cervix	1 (3.2)
**Tumor classification**	
PA colorectal	26 (83.9)
LR colorectal	3 (9.7)
PA non-colorectal	0 (0)
LR non-colorectal	2 (6.4)
**Type of operation**	
PPE	19 (61.3)
PPE with sacrectomy	2 (6.4)
TPE	10 (32.3)
**Operative approach**	
Open	28 (90.3)
Laparoscopic	3 (9.7)
**Radicality**	
R0	21 (67.7)
R1/R2	4/2 (19.3)
Not stated	4 (13)
Operative time (hours), median (range)	5.5 (4-11)
**Stoma**	
End colostomy	20 (64.5%)
End ileostomy	1 (3.2%)
Loop ileostomy	4 (13%)
No	6 (19.3%)
Neoadjuvant CRT	19 (63.3)
Adjuvant chemotherapy	22 (71)

Values are presented as numbers (%) unless otherwise indicated; ASA: American Society of Anesthesiologists; BMI: body mass index; PA: primary advanced; LR: locally recurrent; PPE: posterior pelvic exenteration; TPE: total pelvic exenteration

**Surgery and outcome:** thirty patients underwent 31 surgeries (one patient underwent PPE for a rectal tumor invading the uterus and after 8 months she underwent TPE after a recurrence of the malignancy). The most common primary site of the tumor was the rectum in 24 (77.4%) patients. Twenty-six tumors (83.9%) were primarily advanced and 5 were locally recurrent tumors (colorectal=3, non-colorectal=2). PPE was performed in 19 patients (laparoscopic approach in 3), PPE with sacrectomy in 2, and TPE in ten patients. Six patients in addition to pelvic exenteration had other organ excision (total colectomy=1, right colectomy=2, nephrectomy=2, bilateral adrenalectomy=1) due to synchronous malignancies in the organs excised. The median operative time was 5.5 hours (range of 4-11 hours). The most common colostomy procedure was end colostomy in 20 (64.5%). Most of the patients achieved R0 resection 21(67.7%) surgeries. While in 4 specimens, the resection margin was not stated.

Table 2 demonstrates pathological analysis. Adenocarcinoma was the most common represent pathology in 29(93.5%) patients. In one patient with a recurrent uterine tumor, the pathology was carcinosarcoma and one patient had a recurrent cervical squamous cell carcinoma with direct invasion into the rectum and metastasis to the caecum. The poorly differentiated grade was found in 13(42%) specimens. Inflammatory adhesion rather than tumor invasion into adjacent organs was observed in 5 of 26 patients with PA colorectal. One patient who underwent total colectomy for multiple premalignant colonic polyps with PPE had rectal T3N1, uterine T3bN2, and colonic T2N0 tumors. The median number of lymph nodes excised was 16 (range 0-124). In 12 patients with PA colorectal malignancy, there were no pathologic lymph nodes (N0) found in the specimen. In one patient after neoadjuvant chemoradiotherapy who underwent initially low anterior resection for an anteriorly lying tumor, the pathology report stated that the anterior circumferential margin was compromised so he had TPE with the pathology report showing no malignancy in the specimen removed. The median hospital stay was 6 days (range 2-20 days).

**Table 2:** pathologic data analysis of the resected specimen

Variable	PA colorectal	LR colorectal	LR non-colorectal
	**n (%)**	**n (%)**	**n (%)**
**Tumor type**			
Adenocarcinoma	26 (84)	3 (9.6)	
Squamous carcinoma			1 (3.2)
Carcinosarcoma			1 (3.2)
**Tumor differentiation**			
WD or MD	16 (51.6)	1 (3.2)	1 (3.2)
PD or mucinous	10 (32.2)	2 (6.6)	1 (3.2)
**Tumor status**			
T2	1 (3.2)		
T3	4 (12.7)		
T4	20 (64.5)	1 (3.2)	
Other *	1 (3.2)	2 (6.6)	2 (6.6)
**Node status**			
Positive	13 (42)	1 (3.2)	
Negative	11 (35.2)	1 (0/4) ** (3.2)	2 (6.6)
Other*	1 (3.2)	2 (6.6)	
**LV invasion**			
Present	12 (38.6)	2 (6.6)	1 (3.2)
Absent	12 (38.6)		
Not stated	2 (6.6)	1 (3.2)	1 (3.2)
**Perineural invasion**			
Present	9 (29)	2 (6.6)	1 (3.2)
Absent	13(42.2)		
Not stated	4 (12.9)	1 (3.2)	1 (3.2)
**Mesorectal grading**			
Complete	17 (54.8)		2 (6.6)
Nearly complete	2 (6.6)	1 (3.2)	
Incomplete	4 (12.9)		
Not applied***	5 (16)		

* Other: not stated=1, recurrent residual=1, carcinosarcoma=1, squamous cell carcinoma=1, no malignancy=1; **: zero out of 4 LN (lymph node); ***not applied: sigmoid tumor, recurrent tumor; WD: well differentiated; MD: moderately differentiated; PD: poorly differentiated; PA: primary advanced; LR: locally recurrent; LV: lympho-vascular

The overall complication rate was 67.7%. Table 3 shows the postoperative outcome and follow-up data. Clavien-Dindo grade I and II complications occurred in 6 (26%) and 7 (30.4%), respectively. This included pulmonary, urinary tract, and wound infections, and anastomotic leaks that were managed conservatively. Grade III b (anastomotic leak) occurred in 1 (3.3%) patient that treated by pelvic wash and drain insertion. Four patients (13.3%) presented grade IV complications, pulmonary embolism, and myocardial infarction, requiring admission to the intensive care unit (ICU). In the hospital, mortality occurred in 5 (16.7%) patients (pulmonary embolism = 3, myocardial infarction = 1, and anastomotic leak with sepsis = 1). Six patients were readmitted to the hospital within 30 days after the surgery, 2 for dehydration, 2 for wound infection, 1 for entero-cutaneous fistula, and one for separation of anastomosis. All were managed non-operatively.

**Table 3:** operative and postoperative outcome and follow-up results

Characteristics	Value
Hospital stays (days), median (range)	6 (2-20)
Complications (%)	21 (67.7)
Clavien-Dindo grade I	6 (26)
Clavien-Dindo grade II	7 (30.4)
Wound infection and discharge	4 (19)
Urinary tract infection	1 (4.8)
Pneumonia	2 (9.5)
**Clavien-Dindo grade**	
Grade IIIa	0
Grade IIIb	
Anastomotic leak	1 (4.3)
Clavien-Dindo grade IV	4 (17.4)
Pulmonary embolism	3 (14.3)
Myocardial infarction	1 (4.8)
Clavien-Dindo grade V	
Death	5 (21.7)
In hospital mortality, n (%)	5 (16.7)
Follow-up, n (%)	25 (83.3)
Alive, disease-free	10 (33.3)
Alive with disease	3 (10)
Died of disease recurrence	8 (26.7)
Lost to follow up and alive	4 (13.3)
Readmission, n (%)	6 (20)
**Readmission cause (%)**	
Entero-cutaneous fistula	1 (16.7)
Dehydration	2 (33.3)
Wound infection	2 (33.3)
Anastomosis disruption	1 (16.7)

**Figure 1:**
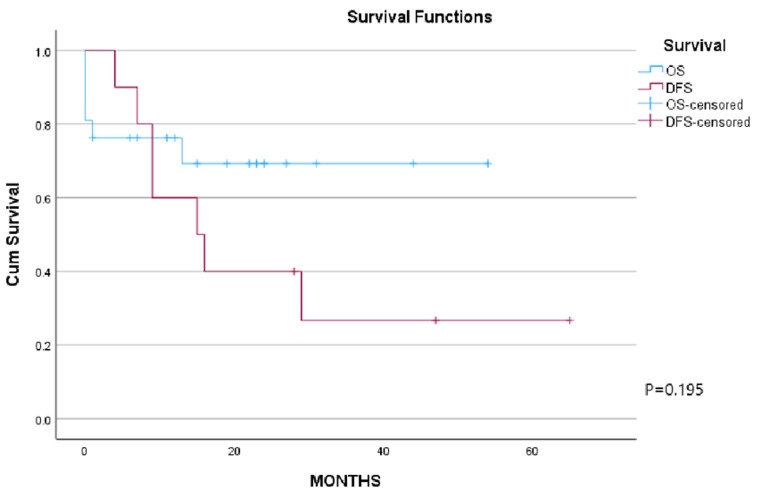
Kaplan-Meier survival analysis curves, overall survival (OS) in blue and disease-free survival (DFS) in red

**Figure 2:**
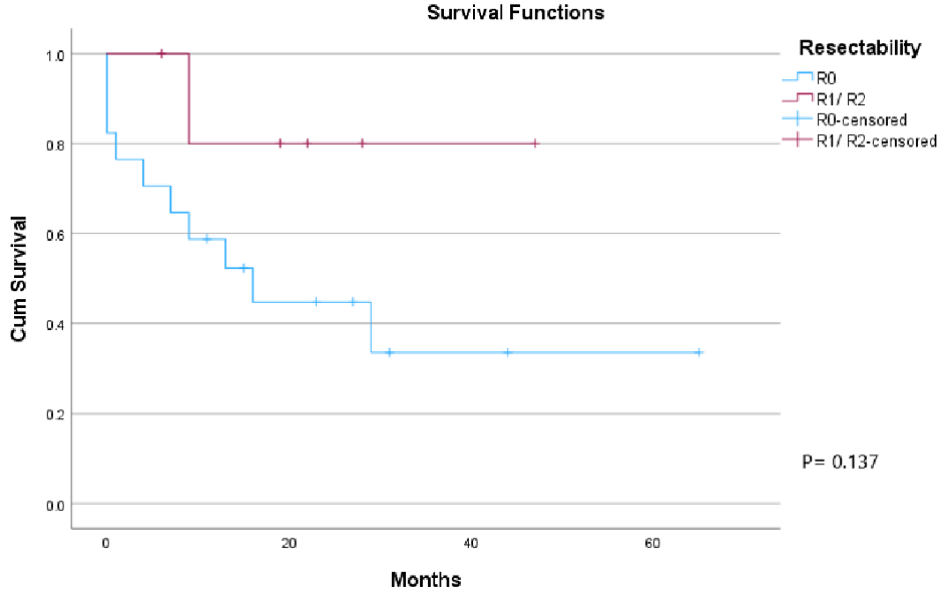
Kaplan-Meier survival analysis curves, overall survival in R0 vs R1 R2 resection, R0 in blue, R1/ R2 in red

**Survival outcome:** over a median follow-up period of 23 months (range 6-65 months) 17 (56.7%) patients died. Five (16.1%) patients died within the primary hospital stay and 8 (26.7%) patients died of disease recurrence within 4-30 months. The remaining 13 (43.3%) patients are alive of whom 10 (33.3%) are disease-free (Figure 1). In patients with involved resection margins (R1/R2), five patients (83.3%) are alive at the last follow-up, of whom 3 patients are disease free. Regarding patients with R0 resection, six (28.6%) patients died of the disease recurrence and seven (33.3%) patients are alive of whom 6 patients are disease-free (Figure 2).

## Discussion

Pelvic exenteration procedure is considered one of the most complex surgical operations. It is defined as multi-visceral radical surgery to cure primary or recurrent pelvic malignancies. This may include colorectal, prostate and bladder, uterine, cervical, and ovarian cancers necessitating complete or partial removal of these pelvic organs, vasculature, musculature, ligaments, or part of the bony pelvic ring [16].

This study assessed the surgical and oncological outcome results after pelvic exenteration for advanced pelvic tumors. This procedure is a complicated surgical intervention with high morbidity and mortality reported in the literature. Morbidity has been reported to range from 37% to 100% and postoperative mortality varies between 0% and 25% [17]. In the current study, the observed overall postoperative complication and mortality rates were 67.7% and 16.7% respectively. The majority (62%) of complications were non-surgical. Surgical complications occurred in 6 patients, early complications occurred in 2 patients, and four patients develop late complications. The major cause of death (80%) was postoperative medical complications (pulmonary embolism and myocardial infarction). Only one patient died of an anastomotic leak and sepsis.

It is common for other organs to be involved in patients with colorectal tumors. Hence, intraoperatively, it is often difficult to differentiate whether the attachment is due to tumor invasion or inflammations [18,19]. The incidence of these adhesions being malignant varies in different studies between 40 and 84% [20]. Whenever possible, the tumor and the involved organ(s) should be resected en bloc, as few series demonstrated an increase in local recurrence rate and compromise patient´s survival in less than radical resection [21,22]. Hunter *et al*. demonstrated a drop in 5-year survival from 61% to 23% when tumors were detached from the organs to which they were attached compared to en bloc resection, in addition, local recurrence increased from 36% to 77% when the tumor was separated from surrounding structures [21]. In the present study, in five out of 31 (16.1%) surgeries, the adherence to other organs was due to inflammatory adhesions that could not be macroscopically differentiated during surgery from malignant adhesions.

The main purpose of exenteration surgery is to achieve an R0 resection. In our series, R0 was achieved in 67.7%, R1 in 12.9%, and R2 in 6.5% of surgeries. These results are comparable to de Nes *et al*. wherein their large series, R1 was present in 19.8% and R2 in 3% after multi-visceral resection for locally advanced rectal cancer [23]. In addition, Bhangu *et al*. in their meta-analysis of 1460 patients with locally recurrent rectal cancer of whom 23% underwent exenteration surgery, 57% underwent an R0 resection, 25% an R1 resection, and 11% an R2 resection. They concluded that patients undergoing R0 resection have the greatest survival advantage following surgery for recurrent rectal cancer. There is a survival advantage for R1 over R2 resection, but there may be no benefit of R2 resection over palliative treatment [17].

Several studies do not differentiate between primary advanced and locally recurrent rectal cancer; both entities are described as one group [24]. It is important to differentiate between these two groups because the 5-year overall survival of patients treated for primary advanced rectal cancer is 40-75% compared to 15-55% in recurrent rectal cancer [25]. A similar difference in local control in favor of primary locally advanced rectal cancer has been reported [26-28].

**Limitations:** this study was limited by the small number of patients included, its retrospective nature and single-center experience. In addition, lack of separation between PA and LR, colorectal, and gynecological cancers resulting in increased heterogeneity owing to the more aggressive nature of LR tumors. Also, minimally invasive surgery was used in three patients only. A prospective multicentric comparative study with a large number with long-term survival is recommended to confirm our result.

## Conclusion

Pelvic exenteration operation remains a complex surgery that requires a multidisciplinary approach. Our study demonstrated that PE is feasible surgery for advanced pelvic malignancies with an overall survival above 40% at a median of 2 years follow-up. However, a huge effort should be made for preoperatively optimization of the patients to reduce morbidity and mortality. Pelvic exenteration can provide an opportunity for good local control and extend long-term survival by achieving an R0 resection.

### What is known about this topic

- *Colorectal and pelvic malignancies can invade other organs and structures in the pelvis which if left untreated can cause debilitating symptoms and high mortality;*
- *In selected patients, some of the locally advanced pelvic cancers can be locally controlled if pelvic exenteration is performed;*
- *The five-year survival rate for patients undergoing pelvic exenteration can reach 75% in advanced specialized centers*.

### What this study adds

- *Pelvic exenteration is visible, although it´s a complex procedure that can be done in a center with limited resources;*
- *This surgery should be considered in a patient with advanced pelvic malignancy when expertise is available*.
